# Mobile health vs. standard care after cardiac surgery: results of The Box 2.0 study

**DOI:** 10.1093/europace/euac115

**Published:** 2022-08-11

**Authors:** Tom E Biersteker, Mark J Boogers, Martin Jan Schalij, Bas B L Penning de Vries, Rolf H H Groenwold, Anouk P van Alem, Arend de Weger, Nicolette van Hof, Roderick W Treskes

**Affiliations:** Department of Cardiology, Leiden University Medical Center, Albinusdreef 2, 2333 ZA Leiden, The Netherlands; Department of Cardiology, Leiden University Medical Center, Albinusdreef 2, 2333 ZA Leiden, The Netherlands; Department of Cardiology, Leiden University Medical Center, Albinusdreef 2, 2333 ZA Leiden, The Netherlands; Department of Clinical Epidemiology and Biomedical Data Sciences, Leiden University Medical Center, Leiden, The Netherlands; Department of Clinical Epidemiology and Biomedical Data Sciences, Leiden University Medical Center, Leiden, The Netherlands; Department of Cardiology, Haaglanden Medisch Centrum, The Hague, The Netherlands; Department of Cardiothoracic Surgery, Leiden University Medical Center, Leiden, The Netherlands; Department of Cardiology, Leiden University Medical Center, Albinusdreef 2, 2333 ZA Leiden, The Netherlands; Department of Cardiology, Leiden University Medical Center, Albinusdreef 2, 2333 ZA Leiden, The Netherlands

**Keywords:** eHealth, mHealth, Remote patient monitoring, Postoperative atrial fibrillation, Cardiac surgical procedures, Coronary artery bypass grafting

## Abstract

**Aims:**

Postoperative atrial fibrillation (POAF) is a common complication of cardiac surgery, yet difficult to detect in ambulatory patients. The primary aim of this study is to investigate the effect of a mobile health (mHealth) intervention on POAF detection after cardiac surgery.

**Methods and results:**

We performed an observational cohort study among 730 adult patients who underwent cardiac surgery at a tertiary care hospital in The Netherlands. Of these patients, 365 patients received standard care and were included as a historical control group, undergoing surgery between December 2017 and September 2018, and 365 patients were prospectively included from November 2018 and November 2020, undergoing an mHealth intervention which consisted of blood pressure, temperature, weight, and electrocardiogram (ECG) monitoring. One physical outpatient follow-up moment was replaced by an electronic visit. All patients were requested to fill out a satisfaction and quality of life questionnaire. Mean age in the intervention group was 62 years, 275 (70.4%) patients were males. A total of 4136 12-lead ECGs were registered. In the intervention group, 61 (16.7%) patients were diagnosed with POAF vs. 25 (6.8%) patients in the control group [adjusted risk ratio (RR) of POAF detection: 2.15; 95% confidence interval (CI): 1.55–3.97]. *De novo* atrial fibrillation was found in 13 patients using mHealth (6.5%) vs. 4 control group patients (1.8%; adjusted RR 3.94, 95% CI: 1.50–11.27).

**Conclusion:**

Scheduled self-measurements with mHealth devices could increase the probability of detecting POAF within 3 months after cardiac surgery. The effect of an increase in POAF detection on clinical outcomes needs to be addressed in future research.

**What’s new?**

- The use of mobile health (mHealth) is observed to increase the probability of detecting postoperative atrial fibrillation.
- The use of mHealth is also observed to decrease visits to the emergency department.
- The mHealth participants adhere well to scheduled self-measurements, although providing technological support is advised.
- Travel distance to the hospital was observed to have no effect on patient’s mHealth satisfaction.

## Introduction

After cardiac surgery, patients are at risk of developing adverse events with a potentially permanent adverse impact on quality of life. Cardiac tamponade, ischaemic stroke, sternal wound infection, heart failure, and postoperative atrial fibrillation (POAF) are frequently diagnosed and treated during the admission period.^1^ The POAF has an incidence of approximately 35%, peaking at Days 2 and 3 after cardiac surgery.^2^ Of all POAF, 15% is diagnosed 7 or more days after cardiac surgery.^3^ The existence of short paroxysms of atrial fibrillation (AF) and the possibility of asymptomatic AF are complicating factors in AF detection. Early diagnosis is important, as untreated AF is associated with an increased risk for transient ischaemic attacks (TIAs) and ischaemic stroke.^4^ Therefore, evaluating new methods to increase the detection rate of symptomatic and asymptomatic AF is important.

Over the past decade, the number of smartphone-compatible wearables allowing remote monitoring [mobile health (mHealth)] increased exponentially. Via mHealth, patients can be instructed from a distance, and therapeutic regimens can be altered. Current European guidelines discuss the possibility of mHealth for early detection of disease.^5,6^ In cardiovascular outpatient care, providers and patients are positive toward the potential mHealth holds,^7,8^ emphasizing an increase in patient engagement and empowerment, but little research has been performed to study the use of mHealth devices in the follow-up of postcardiac surgery patients. There are numerous devices for the detection of AF, based on single-lead electrocardiogram (ECG) or photophlethysmography. Several studies have assessed the clinical effectiveness of available mHealth devices, and a recent systematic review demonstrated these devices to be a viable option to increase the detection rate of AF.^9^ Therefore, the primary aim of this study was to investigate the effect of an mHealth intervention on POAF detection after cardiac surgery. The secondary purpose was to assess the effect of mHealth on readmission, emergency department (ED) visits, and (unplanned) outpatient clinic visits.

## Methods

### Study design

The Box 2.0 was an observational cohort study with a prospective intervention group and a historical control group for comparison. It was conducted at the department of cardiothoracic surgery of the Leiden University Medical Center (LUMC), The Netherlands, between November 2018 and February 2021, and patients were consecutively screened and enrolled. The study was registered under NCT03690492 (ClinicalTrials.gov) and NL65959.058.18 (ToetsingOnline.nl). The Box 2.0 was based on The Box, a randomized controlled trial (RCT) in myocardial infarction patients.^10^ The study complies with the Declaration of Helsinki, and was approved by the ethics committee.

Adult patients, who underwent coronary artery bypass grafting, valve reconstruction or replacement, aortic root surgery, Dor or Morrow procedure, cardiac tumour removal, or any other cardiac surgery requiring sternotomy, including concomitant rhythm surgery, were eligible for enrolment. The exclusion criteria were: pregnancy, incapacitation, ventricular septal rupture, mechanical support at the time of surgery, implantation of a ventricular assist device, and emergency cardiac surgery defined as a score 1 or 2 at the Interagency Registry for Mechanically Assisted Circulatory Support scale. In order to prevent selection bias, patients who declined to take part in the mHealth intervention were also included in the intervention group.

A detailed description of the study design has been published previously.^11^

### Control group

Patients who underwent cardiac surgery at the LUMC between December 2017 and September 2018 were included as a historical control group. Standard follow-up consisted of two physical outpatient clinic visits at the LUMC; 2 weeks and 3 months after discharge. At the 2-week outpatient visit, the sternal wound was examined and a 12-lead, 10 s ECG was made. At 3 months, another ECG was made, and a laboratory test and transthoracic echocardiogram were performed. No remote monitoring was performed. Patients were advised to visit the hospital or general practitioner in case of symptoms of possible AF.

### Intervention group

Intervention group patients were recruited between November 2018 and November 2020. Eligible patients were approached at the outpatient clinic 4–6 weeks before surgery, or on the ward, 1–5 days before surgery or at minimum 3 days after surgery. Patients received oral and written information about the study, and were given at least 24 h to consider participation. All study participants signed the informed consent form before discharge. Recruitment was done by a nurse practitioner (NP), who also conducted the outpatient clinic visits. To assure all eligible patients were approached with study information and informed consent forms, the study team could review the weekly schedule of the thoracic surgery department, and a weekly meeting with this department was organized.

Follow-up in the intervention group consisted of remote monitoring via ‘The Box’. Furthermore, the first outpatient clinic visit at 2 weeks was replaced by an electronic visit (eVisit) via a secured video connection using specifically developed medical software (Webcamconsult, Bergen op Zoom, The Netherlands). The eVisit consisted of the same patient interview as the standard outpatient clinic contact moment and was performed by the same NP. The sternum wound and, if applicable, vasectomy wound were also examined during this eVisit. This eVisit was also available to intervention group patients who declined to take mHealth measurements. The 3-month outpatient clinic visit was identical to the 3-month visit in the control group, and marked the end of follow-up.

### The Box

Patients who consented to participate received a box containing an activity tracker, blood pressure (BP) monitor, thermometer, and a weight scale (all Withings, Issy les Moulineaux, France), as well as two mobile ECG devices: a single-lead I ECG monitor (Alivecor, Mountain View, CA, USA) and an EASI-derived ECG monitor (CardioSecur; Personal MedSystems, Frankfurt, Germany). These devices are shown in Supplementary material online, *Appendix*. However, patients with a cardiac implantable electronic device (CIED) did not receive the Cardiosecur, as these patients were remotely monitored through their CIED. Patients were also to be handed out a pulse-oximeter, which could not be included in The Box due to logistical issues during the COVID-19 pandemic. This is a deviation from the previously published protocol.^11^

The Box 2.0 was handed out before discharge from the LUMC; required mobile applications were installed by eHealth technicians, if necessary. A helpdesk was available throughout the duration of each patients’ participation in the study, to assist with technical issues. Patients who did not own a smartphone were equipped with a loan device free of charge. To warrant the privacy of all study participants, patients were provided with an @hlc.nl email address based on a randomly generated code as the individual’s login name, combined with a randomly generated password. The @hlc.nl domain is owned and maintained by the LUMC, and the data are stored on LUMC servers. Online data from Withings devices and the Cardiosecur were accessed via the Application Programming Interface (API; Withings), or via the protected online dashboard (Cardiosecur). The Withings API allowed all device data to be automatically imported to the electronic medical records of the LUMC, via a protected authentication protocol (OAUTH2).

Patients were requested to record BP, weight, temperature, step count, and single-lead ECG daily for the first 2 weeks, regardless of symptoms. From 2 weeks until the end of follow-up, all measurements were reduced to thrice a week. Additionally, patients were requested to record a Cardiosecur ECG weekly, regardless of symptoms. In case of a potential rhythm disturbance, defined as either the presence of symptoms (most notably: palpitations) or an AF alert by the single-lead ECG device, patients were requested to obtain another Cardiosecur ECG.

At the end of the follow-up period, patients were requested to return the Cardiosecur and, if applicable, the provided smartphone. The other mHealth devices were gifted to all participants. Whenever a patient stopped using The Box, they were asked for their rationale.

### Monitoring of mHealth data

All sent-in device data were reviewed by the NP at least twice weekly. In case of data irregularities, triggered by an automated alarm, the NP contacted patients within 2 business days. An overview of data irregularities has been published previously.^11^ Based on these irregularities, medication could be amended or patients could be scheduled for electrical cardioversion or, if necessary, be requested to visit the ED. Patients were phoned by eHealth technicians after 2 weeks of not receiving any mHealth measurement, reminding them of the importance of these measurements.

### Electrocardiogram devices

**Figure 1:**
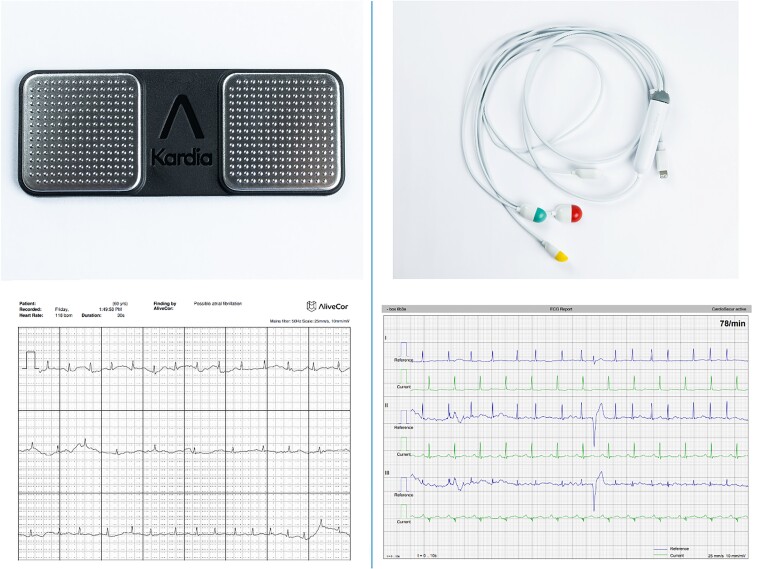
The Alivecor Kardia (top left) and Cardiosecur (top right), and the output of the Alivecor Kardia (bottom left) and Cardiosecur (bottom right).

The Box 2.0 includes two ECG devices, presented in *Figure 1*. The smartphone-compatible single-lead ECG device (Alivecor Kardia) allows patients to record a 30 s lead I ECG. After the measurement is completed, R-R intervals are analysed by the algorithm of the app and the ECG is reported as either normal, potential AF, or undetermined because of noise or an indistinguishable rate or rhythm. The algorithm has a 70–97% sensitivity and 98–99% specificity for the detection of AF.^12^

As baseline artefacts can make it hard to distinguish atrial rhythm disturbances, it was decided to use an EASI-derived 12-lead ECG device (Cardiosecur), first described by Dower *et al*.,^13^ as the main device for POAF detection. This device was advertised to have low baseline noise comparable with Mason–Likar ECGs. The first Cardiosecur ECG was registered by an eHealth technician before discharge. This way, the patient was individually trained to take their own ECG at any time. The NP reviewed each Cardiosecur ECG by logging on to the manufacturer’s online dashboard.

### Primary endpoint

The primary endpoint of this study was POAF detection before the end of follow-up. The AF was defined as an episode of irregular heart rhythm, without detectable *P*-waves, lasting >30 s.^14^ The POAF was diagnosed by the NP, supervised by a consultant cardiologist. In case of POAF, patients were treated as per current European guidelines.^5^

### Secondary endpoints

Secondary endpoints of The Box 2.0 were sternal wound infection and cardiac decompensation after discharge. Sternal wound infection was defined as either fever (temperature >38.5°C), sternal instability, or chest discomfort in combination with either purulent drainage from the sternal wound, or mediastinal widening on radiography. The textbook definition of cardiac decompensation was used.^15^ Other secondary endpoints included all-cause mortality, readmission, ED presentation, major adverse cardiac events (MACEs; a composite endpoint of cardiac tamponade, myocardial infarction, ischaemic stroke, or TIA), patient satisfaction (questionnaire included in the Supplementary material online, *Appendix*), and quality of life [five-level EuroQol five-dimension (EQ-5D-5L)]. The EQ-5D-5L utility value ranges from −1 to 1; scores cannot be compared between patients due to a nonlinear distribution, with a range differing per country.^16^ Therefore, the Dutch Tariff was used.

### COVID-19 and The Box 2.0

During the inclusion period, COVID-19 strongly impacted the capacity of the intensive care unit (ICU) of the LUMC, leading to a decreased number of surgical procedures from March 2020 onwards. This change caused elective surgeries to be cancelled or postponed, including cardiac interventions. This potentially led to selection bias of fitter and younger patients, which is reflected in the baseline characteristics. Moreover, as ICU stay was shortened as much as clinically possible, cardiac surgery patients had a longer stay on the ward, explaining the baseline difference regarding baseline length of stay.

### Statistical analysis

Patient data were analysed according to the intention-to-treat and per-protocol principles, as it was decided to also include patients who declined to take part in the mHealth intervention, but agreed to their data being used for the study. Analyses were performed using R software. For the primary outcome, logistic regression and Cox proportional hazards regression were used. Patients who died before initial discharge and those with permanent AF or AF at discharge were omitted from this analysis. A Kaplan–Meier survival curve of POAF detection was constructed. To correct for possible differences in patient characteristics between study groups, age, length of stay, history of atrial fibrillation, periprocedural ablation, (paroxysms of) POAF during the admission period, and the use of antiarrhythmic medication at discharge were included as covariates in the regression models. Death was a competing event. Age and length of stay were included as natural cubic spline terms based on three inner knots. The exposure variable intervention/control group was included as a stratifying variable to fit different (nonproportional) baseline hazard functions for the exposure groups. All resulting risk ratios (RRs) are ratios of predicted risks, under exposure vs. nonexposure, standardized to the distribution of baseline characteristics of the control group. For the secondary outcome measures, Fisher’s exact tests were performed; patients who died before initial discharge were excluded. As this study was underpowered to detect differences between groups with respect to MACEs and mortality, only descriptive statistics were reported.

## Results

### Recruitment

**Figure 2:**
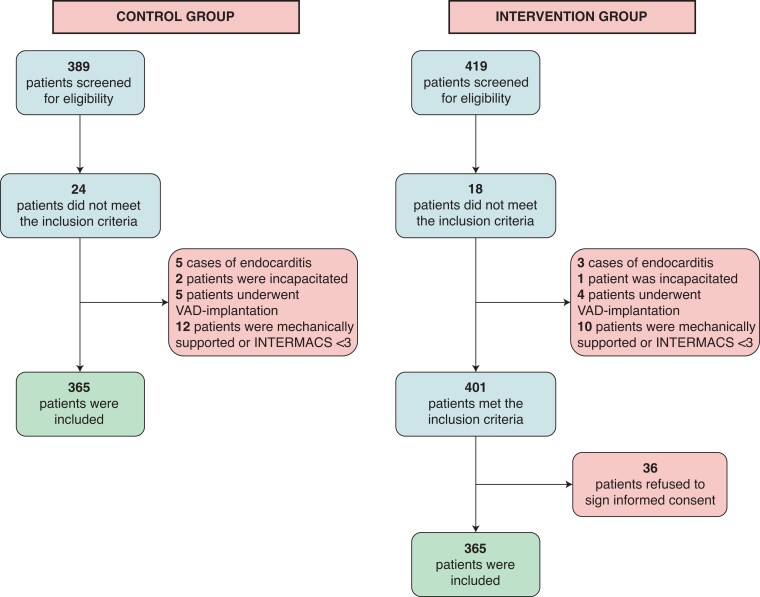
Flow chart of the recruitment process.

After screening 389 patients for eligibility, 365 were included in the control group. After screening 419 patients for eligibility, 365 patients were included in the intervention group. *Figure 2* presents a flow chart of the recruitment process. As this study used an intention-to-treat design, not all included intervention group patients consented to taking home measurements. In total, 319 out of 365 intervention group patients were willing to take home measurements and therefore received The Box 2.0. The other 46 patients did not consent to take home measurements, but consented to the use of their 3-month follow-up data instead. Nine patients (2.8%) of 319 Box participants needed a loaned smartphone, provided by the LUMC.

### Patient characteristics

Mean age of the control group was 66 years, and 278 (76.2%) patients were males. Mean age of the intervention group was 62 years, and 275 (70.4%) patients were males. Fifty-six controls had a history of paroxysmal AF (15.3%) vs. 58 intervention group patients (15.9%, *P* = 0.92); 12 controls had permanent AF (3.3%) vs. 8 intervention group patients (2.2%, *P* = 0.50). The POAF was diagnosed before discharge in 147 controls (40.3%) vs. 128 intervention group patients (35.3%, *P* = 0.17). All baseline characteristics are presented in *Table 1*. Five baseline characteristics showed a statistically significant difference: controls were older (66 vs. 62 years; *P* < 0.0001), more often had a history of smoking (57.3 vs. 49.0%; *P* = 0.03) and diabetes mellitus (25.8 vs. 16.7%; *P* = 0.04), had a shorter hospital stay (7 vs. 8 days; *P* < 0.0001), and were less often on antiarrhythmics (sotalol, amiodarone, flecainide, or dysopyramide) at discharge (61.9 vs. 75.6%; *P* < 0.0001).

**Table 1:** Baseline characteristics

Characteristic	Control (*n* = 365)	Intervention (*n* = 365)	*P*-value
Gender, male (%)	278 (76.2%)	275 (75.3%)	0.86
Age, years (SD)	66.1 (10.5)	62.2 (11.6)	**<0.0001**
BMI, kg/m² (SD)	27.1 (4.1)	26.6 (4.5)	0.14
Travel distance, km (IQR)	16.0 (7.9–19.8)	13.8 (6.0–21.1)	0.50
Travel duration, min (IQR)	21.9 (13.9–28.4)	20.0 (13.1–29.1)	0.35
History of smoking (%)	209 (57.3%)	179 (49.0%)	**0**.**03**
Hypertension (%)	203 (55.6%)	176 (48.2%)	0.05
Hypercholesterolaemia (%)	118 (32.3%)	120 (32.9%)	0.94
Diabetes mellitus (%)	94 (25.8%)	61 (16.7%)	**0**.**004**
COPD (%)	21 (5.8%)	14 (3.8%)	0.30
History of myocardial infarction (%)	92 (25.2%)	97 (26.6%)	0.76
History of CVA/TIA (%)	35 (9.6%)	37 (10.1%)	0.90
Peripheral arterial disease (%)	17 (4.7%)	15 (4.1%)	0.86
History of paroxysmal AF (%)	56 (15.3%)	58 (15.9%)	0.92
Permanent AF (%)	12 (3.3%)	8 (2.2%)	0.50
Cardiac implantable electronic device (%)	15 (4.1%)	17 (4.7%)	0.86
Left ventricle ejection fraction, % (SD)	53.6 (9.9)	55.0 (8.1)	0.16
Urgent operation (%)	90 (24.7%)	94 (25.8%)	0.80
Surgery type (%)			0.10
… CABG	190 (52.1%)	158 (43.3%)	
… Valve	82 (22.5%)	92 (25.2%)	
… CABG + valve	31 (8.5%)	37 (10.1%)	
… Aorta ± valve	43 (11.8%)	62 (17.0%)	
… Morrow procedure	6 (1.6%)	3 (0.8%)	
… Dor procedure	3 (0.8%)	3 (0.8%)	
… Other	10 (2.7%)	10 (2.7%)	
Concomitant AF ablation (%)	36 (9.9%)	38 (10.4%)	0.90
Resternotomy (%)	33 (9.0%)	29 (7.9%)	0.69
Length of hospital stay, days (IQR) [range]	7 (5–9) [2–43]	8 (6–11) [3–83]	**<0.0001**
MACE (%)	29 (7.9%)	23 (6.3%)	0.47
POAF before discharge (%)	147 (40.3%)	128 (35.3%)	0.17
… Median number of days to POAF before discharge (IQR) [range]	2 (2–4) [0–13]	2 (1–3) [0–11]	0.40
Antiarrhythmic drugs at discharge (%)	226 (61.9%)	276 (75.6%)	**0**.**0001**

The bold values indicate a significant difference between research groups.

AF, atrial fibrillation; BMI, body mass index; CABG, coronary artery bypass grafting; COPD, chronic obstructive pulmonary disease; CVA, cerebrovascular accident; IQR, interquartile range; MACE, major adverse cardiac event; POAF, postoperative atrial fibrillation; SD, standard deviation; TIA, transient ischaemic attack.

### Protocol adherence

**Figure 3:**
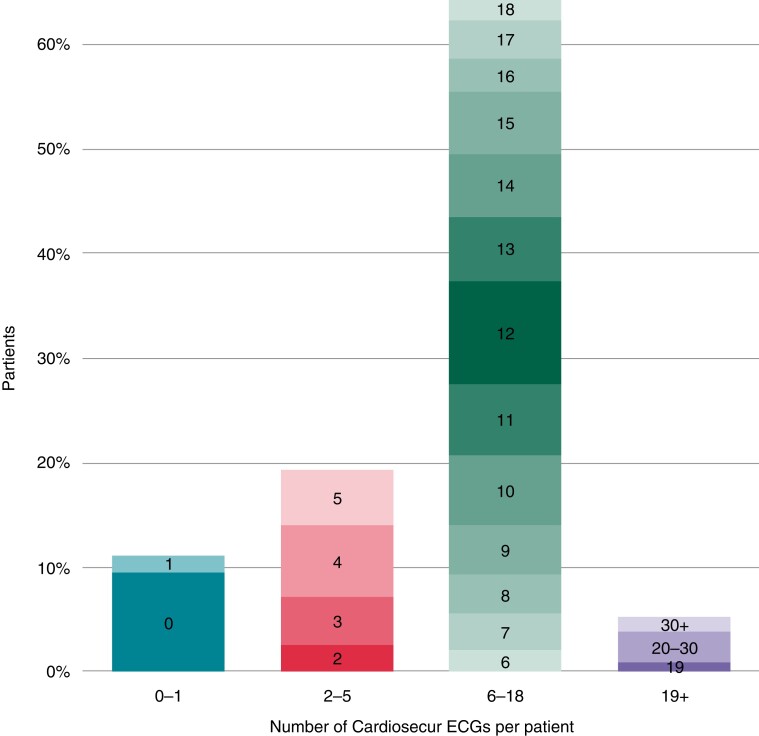
Cardiosecur use. The *x*-axis shows the total number of ECGs made over the course of the 3-month follow-up, which is grouped into four categories: 0–1 ECGs represent patients who tried to use the Cardiosecur to no avail, 2–5 ECGs represent patients who did not adhere to the requested self-measuring frequency, 6–18 ECGs represent adherent patients, and 19+ ECGs represent the patients who registered an ECG (much) more often than requested. The *y*-axis shows the percentage of patients per category, out of all 305 patients with a Cardiosecur. ECG, electrocardiogram.

A total of 74 767 mHealth measurements were recorded by 319 Box patients over the course of 17 926 unique measurement days. Device measurement totals are summarized in Supplementary material online, *Appendix*. Box participants registered a median of 230 measurements during a 92-day follow-up period, averaging 2.5 measurements daily, on a median of 56 out of 92 days [60.9%; interquartile range 40 (43.5%) to 81 (88.0%) days]. Of all measurements, 4136 were ECG registrations made by 276 patients. *Figure 3* shows Cardiosecur usage in more detail. Out of 305 patients with a Cardiosecur, 29 (9.5%) were unable to register an ECG. Furthermore, 59 patients (19.3%) tried to incorporate the Cardiosecur in their follow-up period (2–5 ECGs) and 212 patients (69.5%) successfully did so (≥6 ECGs). Twelve patients (5.2%) used the Cardiosecur more than instructed (>18 ECGs), with one patient registering 73 ECGs.

**Figure 4:**
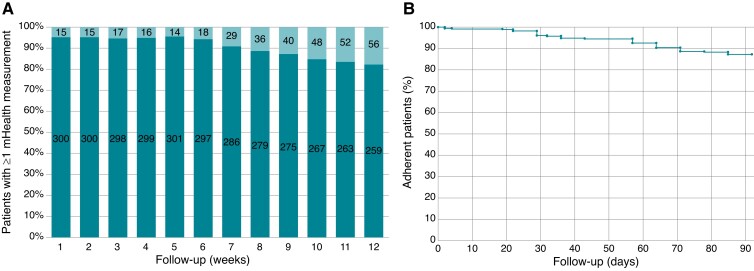
(*A*) mHealth device use during all 12 weeks of follow-up. Out of all 319 intervention group patients with a Box, 4 deceased during follow-up and were excluded from this analysis. Step count measurements were not used for this analysis. (*B*) Kaplan–Meier estimates of nonadherence, defined as ≥21 consecutive days without at least one registered mHealth measurement regarding blood pressure, weight, temperature, or electrocardiogram. Step count measurements were not used for this analysis.

*Figure 4* shows protocol adherence for all Box patients completing follow-up. A total of 41 patients (12.8%) registered no measurements for ≥21 consecutive days and were considered nonadherent. Three patients experienced the home measurements as stressful and stopped using The Box 2.0 at Days 28, 43, and 57, respectively, and one patient was instructed by the general practitioner to stop using The Box 2.0 for unknown reasons. Data of all nonadherent patients were used for the analyses. No patients dropped out of the study.

### Primary endpoint

**Figure 5:**
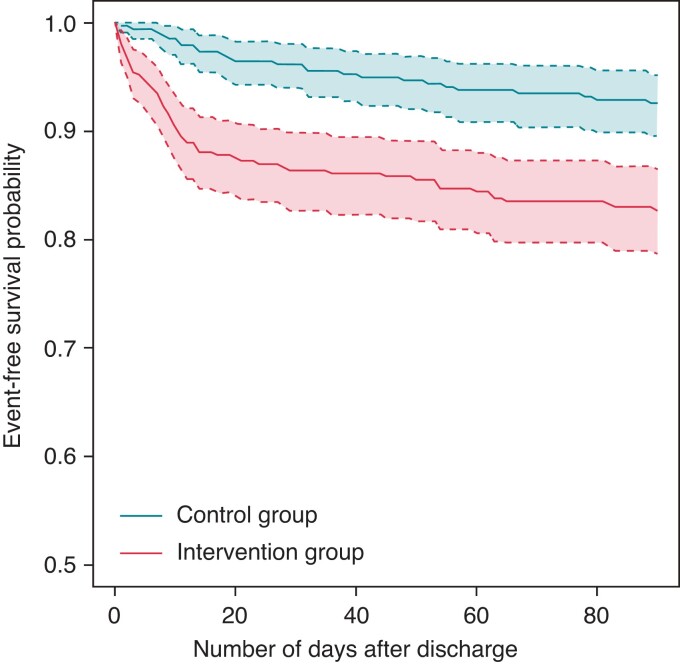
Kaplan–Meier estimates of the cumulative probability of postoperative atrial fibrillation detection over the course of the 3-month follow-up.

In the intervention group, 61 (16.7%) patients were diagnosed with POAF vs. 25 (6.8%) controls. After adjustment, a significant RR of 2.15 [95% confidence interval (CI): 1.55–3.97] was found. For the per-protocol analysis, the adjusted RR was 2.64 (95% CI: 1.77–4.21). A Kaplan–Meier curve for the crude intention-to-treat analysis is shown in *Figure 5*. *Table 2* presents the crude and adjusted RRs of the effect of mHealth on POAF detection vs. standard follow-up. Importantly, when looking into *de novo* AF, 13 cases were found in 199 Box patients without previous AF (6.5%) vs. 4 in 217 non-Box patients (1.8%; adjusted RR 3.94, 95% CI: 1.50–11.27).

**Table 2:** Intention-to-treat and per-protocol analyses for the primary outcome, postoperative atrial fibrillation detection within 3 months after cardiac surgery

	POAF controls	POAF intervention group	Unadjusted RR	95% CI	Adjusted RR^a^	95% CI
*All AF*						
ȃIntention to treat	25 (*n* = 337)	61 (*n* = 352)	2.34	1.53–3.62	2.15	1.50–3.50
ȃPer protocol	28 (*n* = 380)	58 (*n* = 309)	2.55	1.73–4.30	2.64	1.77–4.21
*De novo AF*						
ȃIntention to treat	3 (*n* = 195)	14 (*n* = 221)	4.12	1.46–12.73	2.66	1.02–8.96
ȃPer protocol	4 (*n* = 217)	13 (*n* = 199)	5.09	1.87–15.64	3.94	1.50–11.27

Patients who deceased before initial discharge and those with permanent AF or with AF at discharge have not been added to the analysis.

AF, atrial fibrillation; CI, confidence interval; POAF, postoperative atrial fibrillation; RR, risk ratio.

Corrected for age, length of stay, history of atrial fibrillation, periprocedural ablation, and the use of antiarrhythmic medication at discharge.

### Secondary endpoints

Results of secondary endpoints are presented in *Tables 3 and 4*. In the intervention group, 48 patients (13.4%) had ≥1 unplanned ED visit during follow-up vs. 86 (23.6%) controls [odds ratio (OR) 0.50; 95% CI: 0.34–0.74; *P* = 0.0005). Sternal wound infection was diagnosed in 7 (2.0%) controls and 3 (0.8%) intervention group patients (*P* = 0.22); 13 controls were diagnosed with cardiac decompensation (3.6%) vs. 15 (4.1%) intervention group patients (*P* = 0.85).

**Table 3:** Secondary outcomes

Outcome	Control	Intervention	OR	95% CI	*P*-value
	*n* = 358^a^	*n* = 365^a^			
Sternal wound infection (%)	7 (2.0%)	3 (0.8%)	0.42	0.11–1.62	0.22
Cardiac decompensation (%)	13 (3.6%)	15 (4.1%)	1.13	0.53–2.43	0.85
Readmission (%)	29 (8.1%)	19 (5.2%)	0.62	0.34–1.13	0.17
Unplanned ED visits (%)	86 (24.0%)	48 (13.2%)	0.50	0.34–0.74	**0**.**0005**
	*n* = 358	*n* = 214			
Pre-COVID ED visits (%)	86 (24.0%)	30 (14.0%)	0.52	0.32–0.83	**0**.**004**
	*n* = 336^b^	*n* = 337^b^			
EQ-5D-5L at 3 months	0.82	0.79			0.08
Satisfaction score (SD)	7.9 (1.8)	8.2 (1.5)			**0**.**02**

Unplanned ED visits are reported as a total and are also been specified for the control group patients (*n* = 365) and intervention group patients (*n*) who have completed their follow-up before the COVID-19 pandemic started in The Netherlands in March 2020. The bold values indicate a significant difference between research groups.

95% CI, 95% confidence interval; ED, emergency department; OR, odds ratio; SD, standard deviation.

Deceased patients have not been taken into consideration.

336 controls (94.1%) filled out the questionnaires vs. 337 (93.3%) intervention patients.

**Table 4:** mHealth satisfaction

Intervention	Satisfaction score (SD)		
Box (0–5)	4.14 (0.82)		
eVisit (0–5)	3.70 (1.05)		
	**Appropriate**	**Too many**	**Insufficient**
Measurement frequency	268 (89.9%)	25 (8.4%)	5 (1.7%)
Number of devices	254 (85.2%)	40 (13.4%)	4 (1.3%)

About 298 out of 319 (93.4%) intervention group patients with The Box completed the satisfaction questionnaire at 3 months.

SD, standard deviation.

The EQ-5D-5L and satisfaction questionnaires were completed by 336 (94.1%) controls and 337 (93.3%) significantly more satisfied intervention group patients (*P* = 0.02). Mean EQ-5D-5L scores were 0.79 for controls vs. 0.82 for intervention group patients (*P* = 0.08), indicating no significant difference in quality of life between both groups. Mean patient satisfaction was 8.2 out of 10 for the intervention group and 7.9 for controls (*P* = 0.02). No correlation was found between overall or Box-related satisfaction and living distance to the hospital (*P* = 0.73 and 0.79, respectively).

#### Box satisfaction

On a scale of 1–5, Box and eVisit satisfaction scored 4.1 and 3.7, respectively. Measurement frequency and number of devices were reported to be appropriate by 268 (89.9%) and 254 patients (85.2%), respectively. Five patients (1.7%) would have preferred a higher measurement frequency, and four patients (1.3%) opted for more mHealth devices. However, 25 patients (8.4%) would have preferred a lower measurement frequency, and 40 patients preferred fewer devices (13.4%). Of these patients, 28 (70.0%) reported this was solely due to the Cardiosecur.

### Loss to follow-up, mortality, and major adverse cardiac events

Of all 730 included patients, 21 did not finish the 3-month follow-up. Twelve patients died: 4 (1.1%) of the intervention group and 8 (2.2%) of all controls, of whom 7 (1.9%) died before discharge. Causes of death were: postprocedural endocarditis (*n* = 2), sepsis with multiorgan failure (*n* = 2), atrioventricular rupture, left ventricular hypertrophy with hypovolaemia, and terminal heart failure. Two patients died due to an unknown cause; one died because of out-of-hospital cardiac arrest, 23 days after discharge. In the intervention group, all deaths occurred after discharge. The causes were RV rupture (10 days after discharge), terminal heart failure (62 days), ST-elevation myocardial infarction with papillary muscle rupture (82 days), and death to an unknown cause at home (10 days). Nine patients were lost to follow-up: three intervention group patients and six controls. These patients were all alive at the end of follow-up. No MACEs occurred during follow-up.

## Discussion

### Key findings

This study reports the results of an mHealth intervention to detect POAF after cardiac surgery, in which participants made 4136 ECGs, a median of 11 per patient during a 3-month follow-up period. A significant increase in POAF detection and *de novo* AF detection was observed. Also, a significant decrease of unplanned ED visits was observed, although an NP monitored intervention group patients more closely because of their mHealth data. There was no significant difference in readmission rates, diagnosis of sternal wound infection, or cardiac decompensation.

#### Postoperative atrial fibrillation detection

At baseline, antiarrhythmic drug use differed significantly. This may be explained by updated in-house cardiac surgery protocols from January 2019 onwards, when sotalol became standard medication postprocedurally, potentially decreasing the risk to develop POAF in intervention group patients but not in controls. However, significantly more POAF and *de novo* AF were detected in the intervention group.

Noticeably, the difference in POAF detection between the intervention and control groups is largest in <2 weeks after initial discharge. As Box patients tended to lose motivation after 2 months, shown in *Figure 4*, it could be beneficial to keep the duration of ECG follow-up short to further improve engagement. A 2017 meta-analysis showed that a non-mHealth ECG intervention in cardiac surgery patients led to a POAF incidence rate of 28.3% (95% CI: 23.0–33.6%; six studies, *n* = 1125) within the first 2–4 weeks after discharge.^17^ Finally, a small minority of patients (*n* = 12, 5.2%) used The Box more often than requested. This data deluge may be a challenge in case cardiologists cannot be reimbursed for these extra checks.

#### Emergency department visits

Overcrowding of EDs is a problem worldwide, and associated with worse patient outcomes and increased costs.^18^ Reducing unnecessary ED visits is important, as cardiac complaints are among the most common reasons to visit an ED. The current study observed a significant decrease in unplanned ED visits in intervention group patients, which is hypothesized to be due to an improved patient engagement and empowerment, as has been mentioned in literature before.^7^ In an earlier paper about ‘The Box’, NPs already described to receive fewer questions from Box patients, and these questions to be more on topic compared with those of other patients.^19^ We therefore hypothesize this educational effect to have contributed to the reduction of ED visits in the intervention group, although intervention group patients’ data were checked twice weekly and therefore received additional care.

#### Satisfaction

Overall satisfaction was high in both study groups, but significantly higher in the intervention arm. The Box was overall described as easy to use, and 89.9% of all Box participants found the prescribed measurement frequency to be appropriate. This difference is hypothesized to be caused by improved individualized care (‘patient tailoring’) and an increased insight The Box provided in the patient’s own health status,^19^ but may also be partly explained by The Box largely being a gift and by the fact that the intervention group received additional care.

Although mHealth has been proposed to improve access to healthcare in rural areas,^20^ distance from the LUMC had no effect on overall or Box-related satisfaction. Travel distance to any amenity in The Netherlands is limited, with a mean distance of 1.0 km to the general practitioner, and 4.7 km to the nearest hospital.^21^ Therefore, the median distance of 14 km to the LUMC is often perceived as far by Dutch patients.

### Comparison with literature

Not many studies have looked into the effects of mHealth in follow-up of cardiac surgery patients. In 2016, McElroy *et al*. published pilot results from an RCT involving 27 intervention patients and 416 controls postcardiac surgery, to assess the impact of mHealth on readmission rates.^22^ A weight scale, pulse-oximeter, and heart rate and BP monitor were used. No significant difference was found for readmission (7.4 vs. 9.9%, *P* = 0.65); readmission rates of the current study were 5.2% in the intervention arm vs. 8.1% in controls (*P* = 0.14). No difference in POAF detection was found.

However, plenty of mHealth initiatives have incorporated rhythm monitoring. A recent review of 14 studies using an mHealth intervention for the follow-up of patients with cardiovascular disease found an increased number of AF diagnoses compared with standard care, with ORs varying from 1.54 to 19.16.^9^ This is in line with the current study. No comparative studies were found using mHealth for the detection of sternal wound infection or heart failure after cardiac surgery. A single-arm, single-device study in postcardiac surgery patients with a mean follow-up of 79 days showed patients to stop using the device after 52 days,^23^ a trend that is also observed in the current study.

### Electrocardiogram devices

As anticoagulants are almost always indicated in case of POAF, diagnostic accuracy was very important. As the Alivecor Kardia has been described to be susceptible to signal interference,^24^ which is also reported by the manufacturer, the 12-lead Cardiosecur was chosen as the main ECG device. However, due to the mandatory use of all four leads, using the Cardiosecur was not always easy. A stable connection could not always be warranted, especially in case of a CIED. These technological difficulties impacted patient adherence and satisfaction, as was demonstrated in *Figure 4*; 28 Box patients specifically stated the Cardiosecur to be their only frustration during follow-up. In contrast, provider satisfaction was high. The NPs stated that the output of the Cardiosecur was easy to read and relatively free of noise.

### Strengths and limitations

The main strength of this study is its large intervention group, with 319 postcardiac surgery patients joining the mHealth intervention. Protocol adherence was good, with a high total measurement and ECG count, and a high number of unique measurement days.

The main limitation of our study is its observational pre-post design, although the intention-to-treat design limited the risk of bias as much as possible. However, patients were not matched and as COVID-19 impacted the inclusion of patients after March 2020, risk of selection bias grew. Also, 36 patients with an average age of 74 declined to sign the informed consent form. Finally, the wide-spread applicability is uncertain, as internet and smartphone use in The Netherlands is high compared with other countries.

## Conclusions

This observational cohort study shows that follow-up with mHealth devices could increase POAF detection in postcardiac surgery. The effects on AF complications need to be addressed in further research. Also, this study observed a reduced number of unplanned ED visits in the intervention arm, although an NP reviewed mHealth data twice weekly.

## Supplementary material

Supplementary material is available at *Europace* online.

## Supplementary Material

euac115_Supplementary_Data

## Data Availability

The data that support the findings of this study are available from the corresponding author, upon reasonable request.
