# Supplementary material for: Mobile health vs. standard care after cardiac surgery: results of The Box 2.0 study
Source: Europace. 2022 Aug 11;25(1):49–58. doi: 10.1093/europace/euac115 (PMC9907478; doi:10.1093/europace/euac115)
Supplement: euac115_Supplementary_Data [file euac115_supplementary_data.docx]

**Appendix**

**
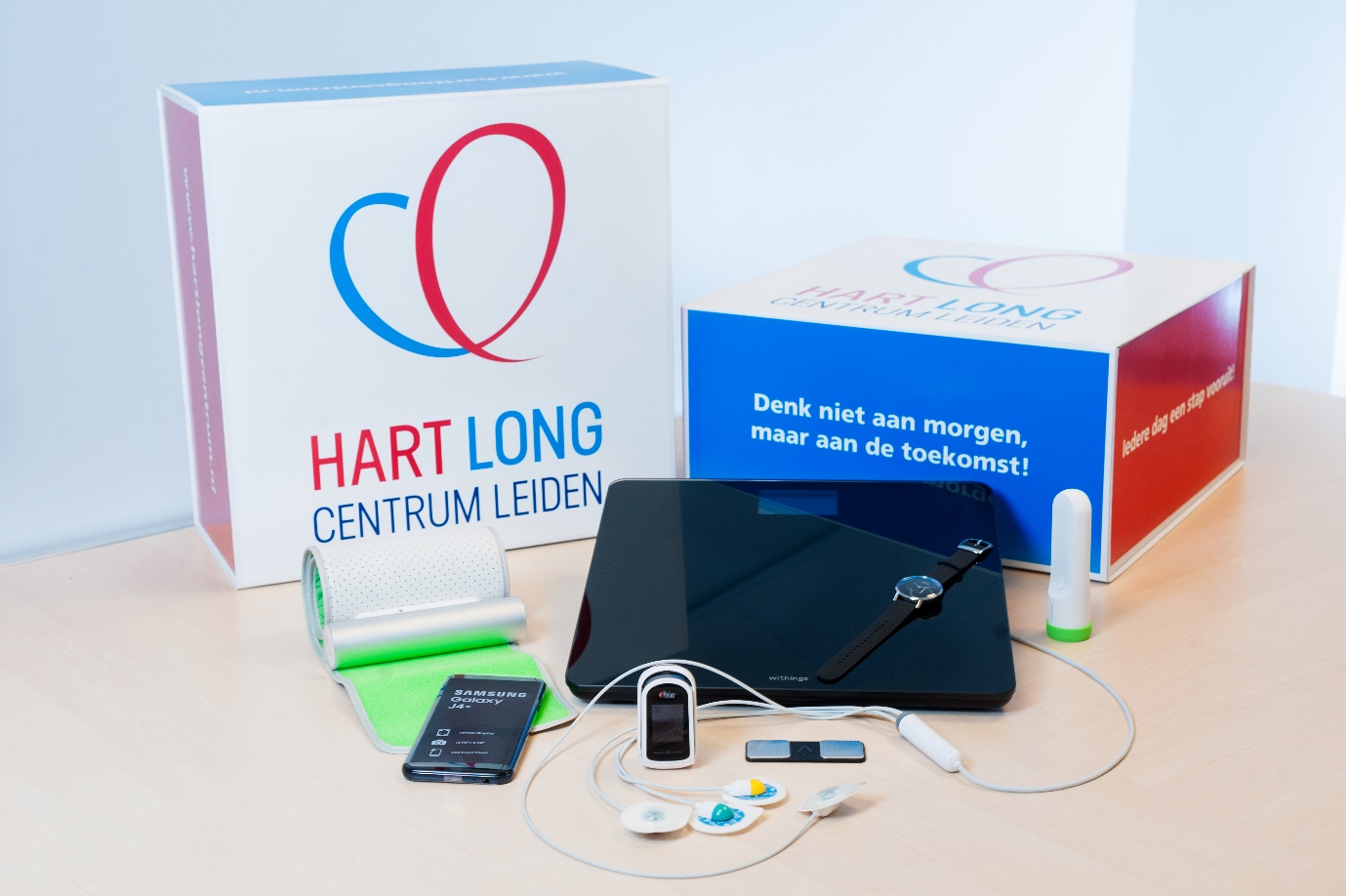
**

*Figure: the Box with all mHealth devices.*

mHealth measurement overview

| **Measurement** | **Total** | **Median** | **IQR** | **Range** |
| --- | --- | --- | --- | --- |
| Blood pressure | 19,463 | 46 | 24 - 76 | 0 - 307 |
| Weight | 16,207 | 47 | 26 - 77 | 0 - 212 |
| Temperature | 12,804 | 33 | 14 - 61 | 0 - 153 |
| Step count days | 22,208 | 89 | 58 - 92 | 0 - 92 |
| ECGs | 4,136 | 11 | 5 - 15 | 0 - 131 |
| Measurement total | 74,767 | 230 | 166 - 295 | 1 - 561 |
| Unique measurement days | 17,926 | 56 | 40 - 81 | 1 - 92 |

*Table: mHealth measurement totals. The maximum number of measurement days is 92, as follow-up stopped after 3 months.*

Patient satisfaction questionnaire

I am satisfied with the quality of healthcare I’ve been provided:

(Dutch: Ik ben tevreden met de kwaliteit van medische zorg die ik ontvang)

- Strongly agree

- Agree

- Neutral

- Disagree

- Strongly disagree

I have easy access to the healthcare providers I need:

(Dutch: Ik heb het gevoel dat ik laagdrempelig contact kan hebben met mijn behandelaars)

- Strongly agree

- Agree

- Neutral

- Disagree

- Strongly disagree

My healthcare providers are good about explaining what’s wrong with me and what needs to be done:

(Dutch: Ik krijg goede uitleg over mijn diagnose en behandeling)

- Strongly agree

- Agree

- Neutral

- Disagree

- Strongly disagree

I think my healthcare providers have everything needed to provide complete medical care:

(Dutch: Ik vind dat het ziekenhuis met voldoende faciliteiten is uitgerust om mij goede zorg te kunnen verlenen)

- Strongly agree

- Agree

- Neutral

- Disagree

- Strongly disagree

I don’t have to wait long for my follow-up appointment:

(Dutch: Ik hoef niet lang te wachten op een vervolgafspraak)

- Strongly agree

- Agree

- Neutral

- Disagree

- Strongly disagree

I am happy with The Box:

(Dutch: Ik ben tevreden met The Box)

- Strongly agree

- Agree

- Neutral

- Disagree

- Strongly disagree

I am happy with the eVisit:

(Dutch: Ik ben tevreden met het webcamconsult)

- Strongly agree

- Agree

- Neutral

- Disagree

- Strongly disagree

The Box provided me with more insight in my state of health:

(Dutch: Met The Box heb ik meer inzicht gekregen in mijn gezondheidssituatie)

- Strongly agree

- Agree

- Neutral

- Disagree

- Strongly disagree

**Text tables**

| **Characteristic** | **Control (n=365)** | **Intervention (n=365)** | **P value** |
| --- | --- | --- | --- |
| Gender, male (%) | 278 (76.2%) | 275 (75.3%) | 0.86 |
| Age, years (SD) | 66.1 (10.5) | 62.2 (11.6) | **< 0.0001** |
| BMI, kg/m² (SD) | 27.1 (4.1) | 26.6 (4.5) | 0.14 |
| Travel distance, km (IQR) | 16.0 (7.9-19.8) | 13.8 (6.0-21.1) | 0.50 |
| Travel duration, minutes (IQR) | 21.9 (13.9-28.4) | 20.0 (13.1-29.1) | 0.35 |
| History of smoking (%) | 209 (57.3%) | 179 (49.0%) | **0.03** |
| Hypertension (%) | 203 (55.6%) | 176 (48.2%) | 0.05 |
| Hypercholesterolemia (%) | 118 (32.3%) | 120 (32.9%) | 0.94 |
| Diabetes Mellitus (%) | 94 (25.8%) | 61 (16.7%) | **0.004** |
| COPD (%) | 21 (5.8%) | 14 (3.8%) | 0.30 |
| History of myocardial infarction (%) | 92 (25.2%) | 97 (26.6%) | 0.76 |
| History of CVA/TIA (%) | 35 (9.6%) | 37 (10.1%) | 0.90 |
| Peripheral arterial disease (%) | 17 (4.7%) | 15 (4.1%) | 0.86 |
| History of paroxysmal AF (%) | 56 (15.3%) | 58 (15.9%) | 0.92 |
| Permanent AF (%) | 12 (3.3%) | 8 (2.2%) | 0.50 |
| Cardiac implantable electronic device (%) | 15 (4.1%) | 17 (4.7%) | 0.86 |
| Left ventricle ejection fraction, % (SD) | 53.6 (9.9) | 55.0 (8.1) | 0.16 |
| Urgent operation (%) | 90 (24.7%) | 94 (25.8%) | 0.80 |
| Surgery type (%) |  |  | 0.10 |
| … CABG | 190 (52.1%) | 158 (43.3%) |  |
| … Valve | 82 (22.5%) | 92 (25.2%) |  |
| … CABG + Valve | 31 (8.5%) | 37 (10.1%) |  |
| … Aorta +/- Valve | 43 (11.8%) | 62 (17.0%) |  |
| … Morrow procedure | 6 (1.6%) | 3 (0.8%) |  |
| … DOR procedure | 3 (0.8%) | 3 (0.8%) |  |
| … Other | 10 (2.7%) | 10 (2.7%) |  |
| Concomitant AF ablation (%) | 36 (9.9%) | 38 (10.4%) | 0.90 |
| Re-sternotomy (%) | 33 (9.0%) | 29 (7.9%) | 0.69 |
| Length of hospital stay, days (IQR) [Range] | 7 (5-9) [2-43] | 8 (6-11) [3-83] | **< 0.0001** |
| MACE (%) | 29 (7.9%) | 23 (6.3%) | 0.47 |
| POAF before discharge (%) | 147 (40.3%) | 128 (35.3%) | 0.17 |
| … Median number of days to POAF before discharge (IQR) [Range] | 2 (2-4) [0-13] | 2 (1-3) [0-11] | 0.40 |
| Antiarrhythmic drugs at discharge (%) | 226 (61.9%) | 276 (75.6%) | **0.0001** |

*Table 1: baseline characteristics*

| All AF |  |  |  |  |  |  |
| --- | --- | --- | --- | --- | --- | --- |
|  | **POAF controls (n=337)** | **POAF intervention grp (n=352)** | **Unadjusted RR** | **95% CI** | **Adjusted RR*** | **95% CI** |
| **Intention-to-treat** | 25 | 61 | 2.34 | 1.53 - 3.62 | 2.15 | 1.50 - 3.50 |
|  | **POAF controls (n=380)** | **POAF intervention grp (n=309)** |  |  |  |  |
| **Per protocol** | 28 | 58 | 2.55 | 1.73 - 4.30 | 2.64 | 1.77 – 4.21 |
| De novo AF |  |  |  |  |  |  |
|  | **POAF controls (n=195)** | **POAF intervention grp (n=221)** | **Unadjusted RR** | **95% CI** | **Adjusted RR*** | **95% CI** |
| **Intention-to-treat** | 3 | 14 | 4.12 | 1.46 - 12.73 | 2.66 | 1.02 - 8.96 |
|  | **POAF controls (n=217)** | **POAF intervention grp (n=199)** |  |  |  |  |
| **Per protocol** | 4 | 13 | 5.09 | 1.87 - 15.64 | 3.94 | 1.50 - 11.27 |

*Table 2: intention-to-treat and per-protocol analyses for the primary outcome, POAF detection within three months after cardiac surgery. Patients who deceased before initial discharge, and those with permanent AF or with AF at discharge have not been added to the analysis.*

**Corrected for age, length of stay, history of atrial fibrillation, periprocedural ablation and the use of antiarrhythmic medication at discharge.*

| **Outcome** | **Control (n=358*)** | **Intervention (n=365*)** | **OR** | **95% CI** | **P value** |
| --- | --- | --- | --- | --- | --- |
| Sternal wound infection (%) | 7 (2.0%) | 3 (0.8%) | 0.42 | 0.11 - 1.62 | 0.22 |
| Cardiac decompensation (%) | 13 (3.6%) | 15 (4.1%) | 1.13 | 0.53 - 2.43 | 0.85 |
| Readmission (%) | 29 (8.1%) | 19 (5.2%) | 0.62 | 0.34 - 1.13 | 0.17 |
| Unplanned ED-visits (%) | 86 (24.0%) | 48 (13.2%) | 0.50 | 0.34 - 0.74 | **0.0005** |
|  | **Control (n=358)** | **Intervention (n=214)** |  |  |  |
| *Pre-COVID ED-visits (%)* | 86 (24.0%) | 30 (14.0%) | 0.52 | 0.32 - 0.83 | **0.004** |
|  | **Control (n=336†)** | **Intervention (n=337†)** |  |  |  |
| EQ5D at three months | 0.82 | 0.79 |  |  | 0.08 |
| Satisfaction score (SD) | 7.9 (1.8) | 8.2 (1.5) |  |  | **0.02** |

*Table 3a: secondary outcomes. ED = Emergency Department; OR = Odds Ratio; 95% CI = 95% Confidence Interval. Unplanned ED visits are reported as a total, and are also been specified for the control group patients (n=365) and intervention group patients (n=) who have completed their follow-up before the COVID-19 pandemic started in The Netherlands, in March 2020.*

**Deceased patients have not been taken into consideration. † 336 controls (94.1%) filled out the questionnaires, versus 337 (93.3%) intervention patients.*

| **Intervention** | **Satisfaction score (SD)** |  |  |
| --- | --- | --- | --- |
| Box (0-5) | 4.14 (0.82) |  |  |
| eVisit (0-5) | 3.70 (1.05) |  |  |
|  | **Appropriate** | **Too many** | **Insufficient** |
| Measurement frequency | 268 (89.9%) | 25 (8.4%) | 5 (1.7%) |
| Number of devices | 254 (85.2%) | 40 (13.4%) | 4 (1.3%) |

*Table 3b: mHealth satisfaction; 298 out of 319 (93.4%) intervention group patients with The Box completed the satisfaction questionnaire at three months.*

**Figures**


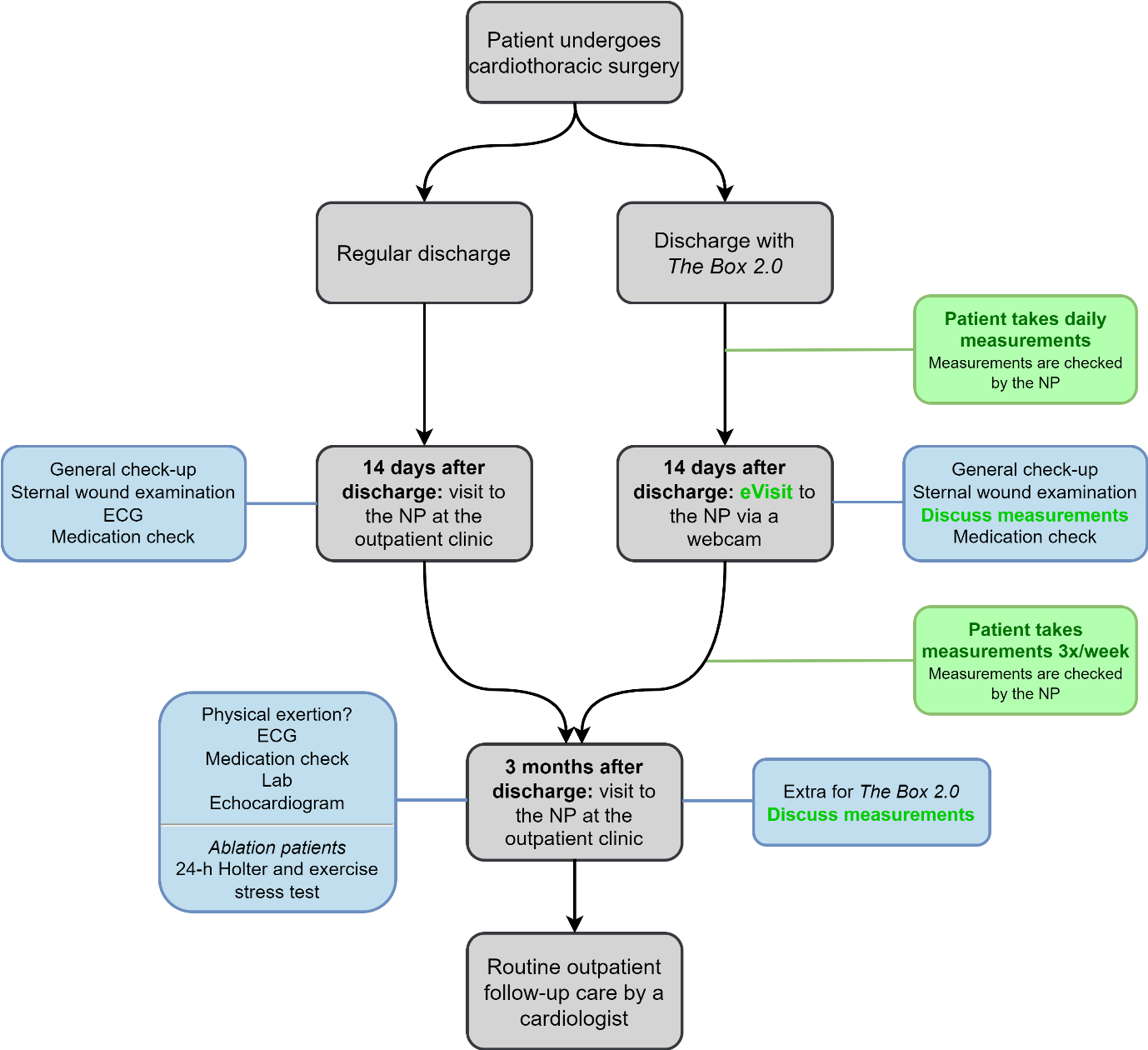


*
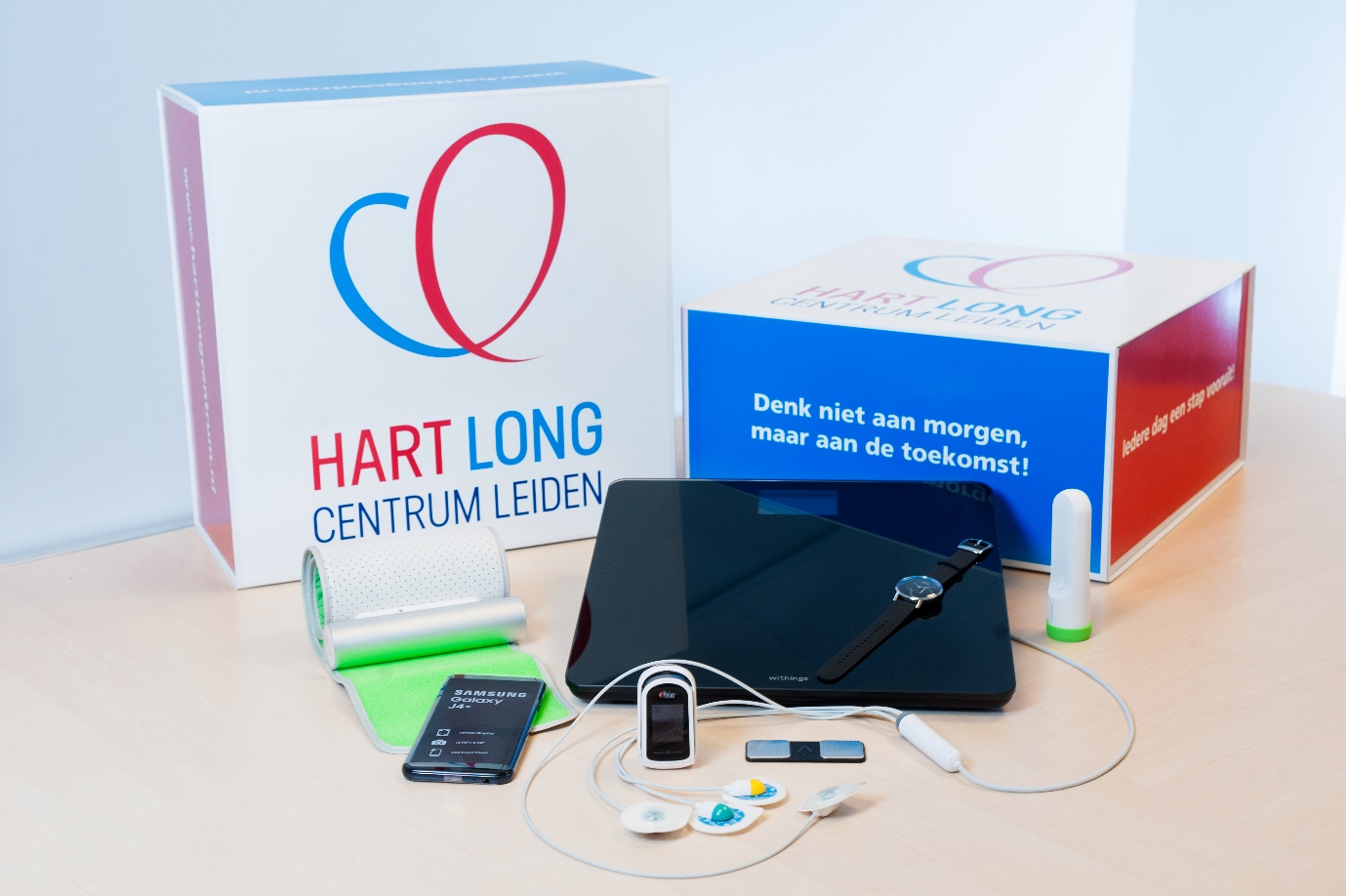
*


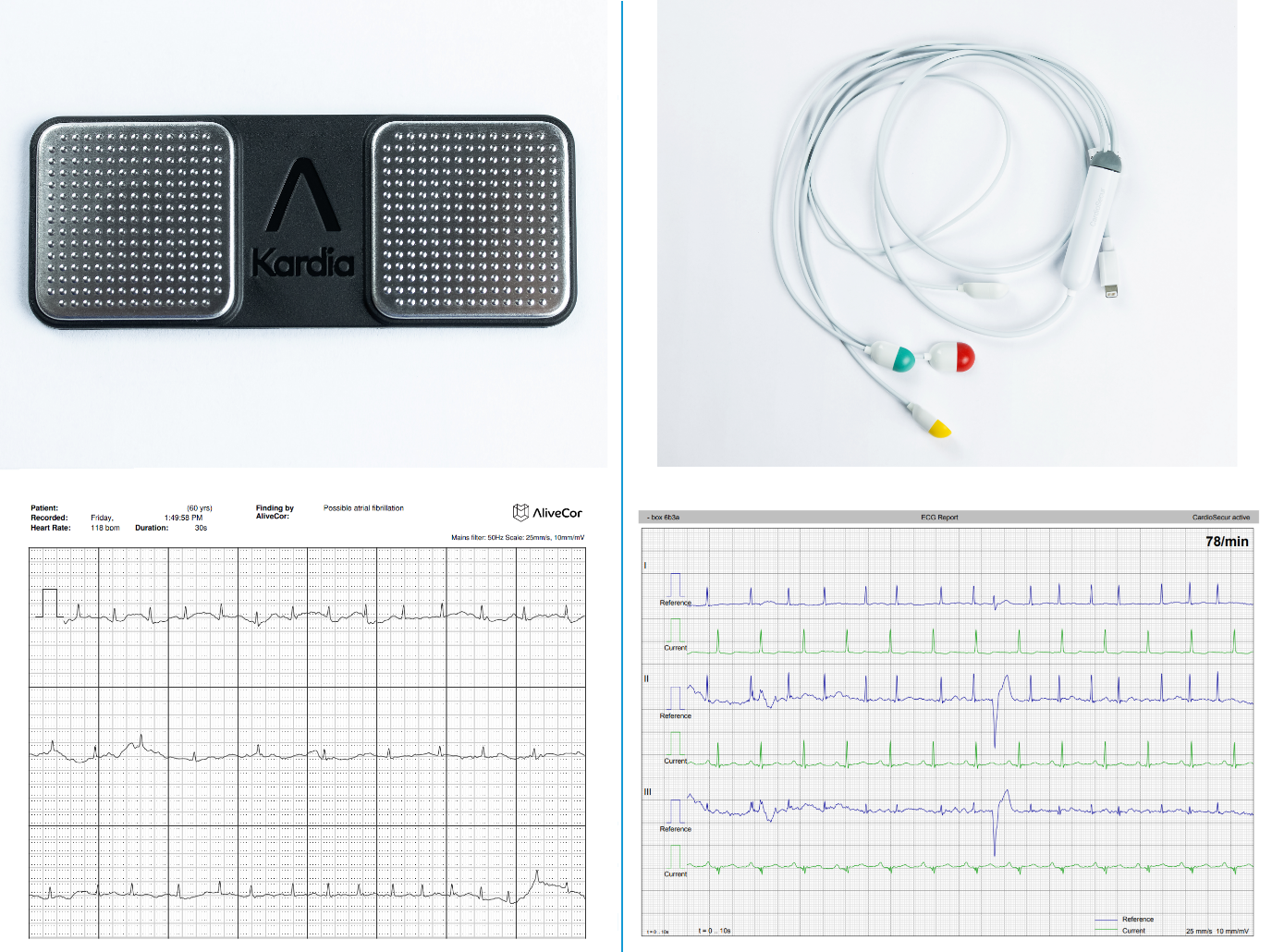


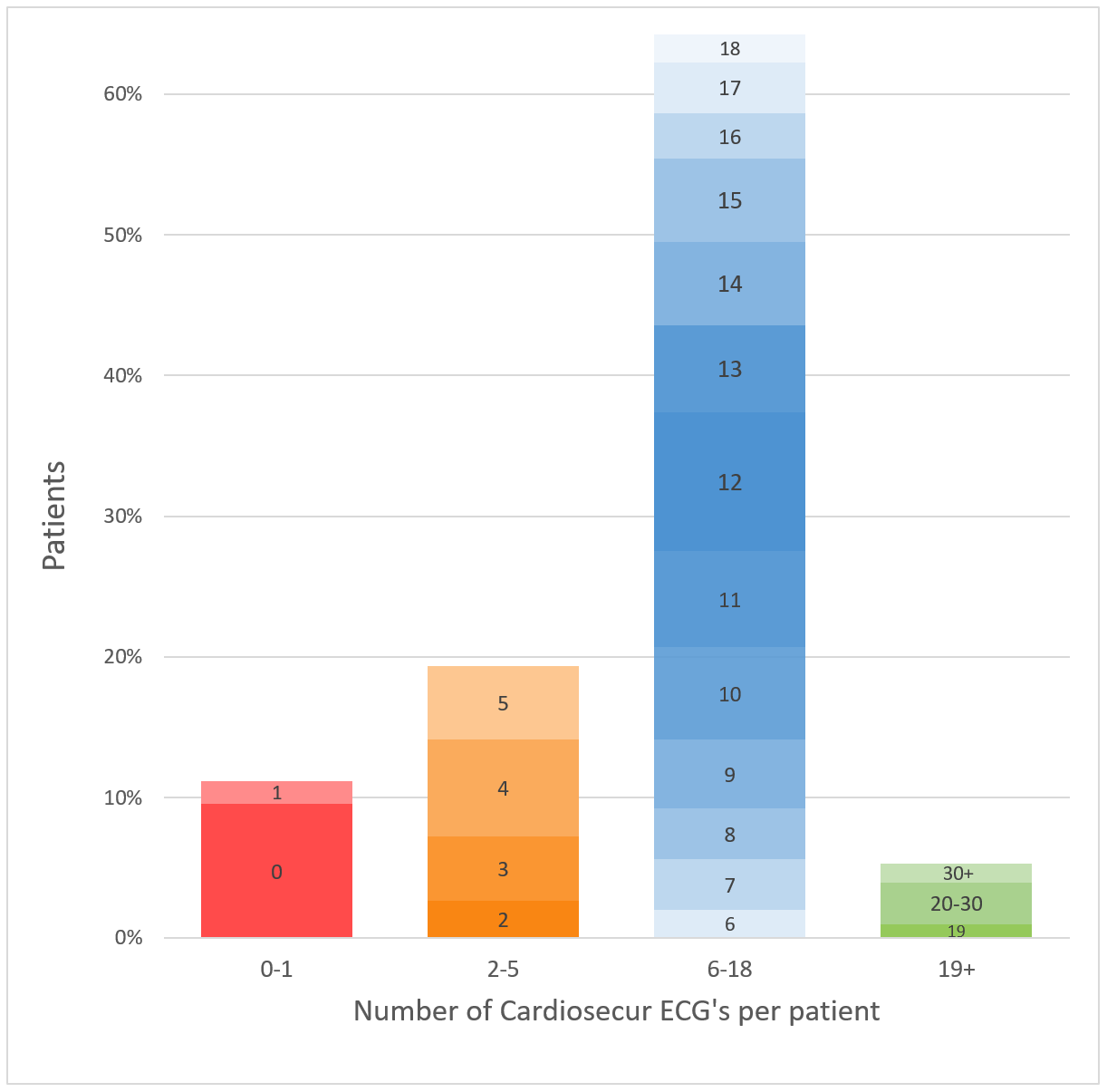


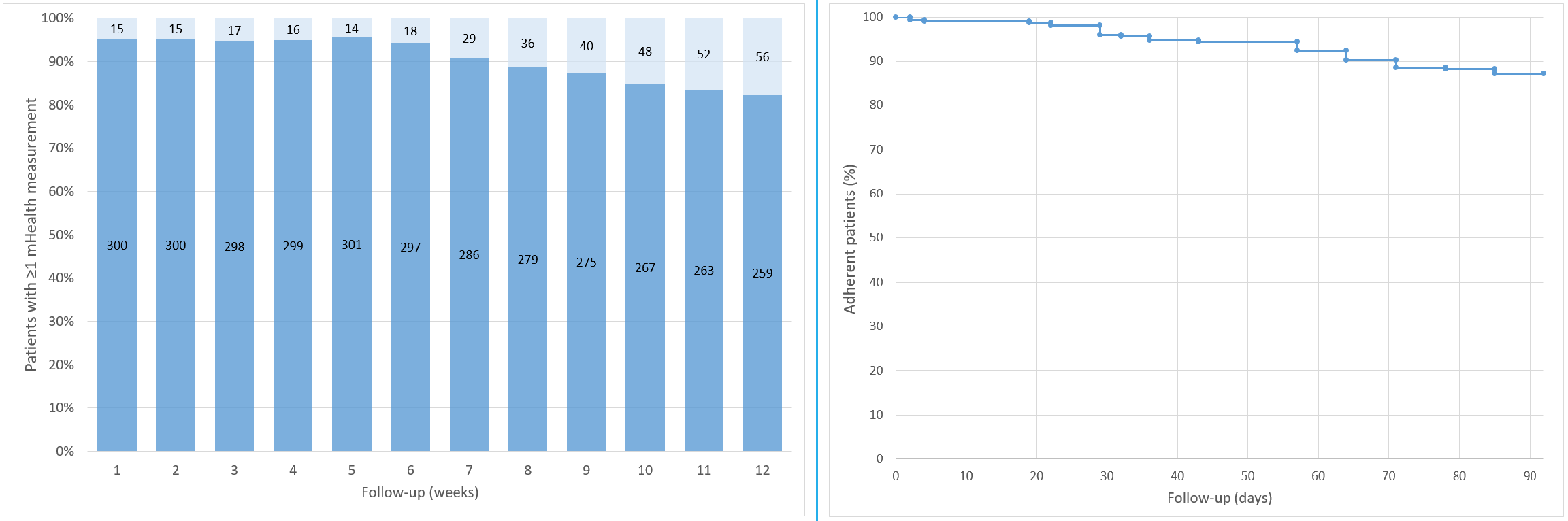


*
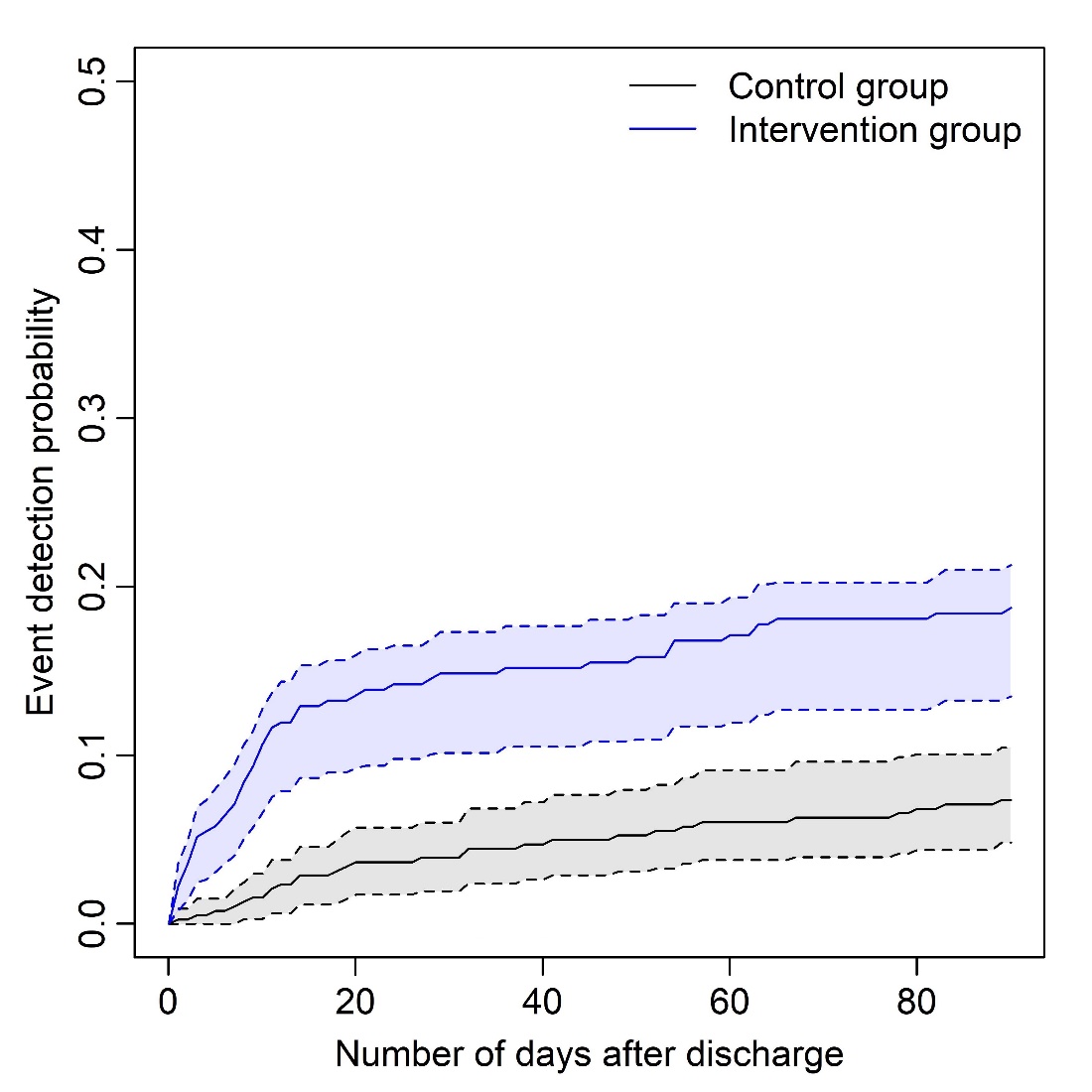
*
